# Diruthenium Paddlewheel Complexes Attacking Proteins: Axial versus Equatorial Coordination

**DOI:** 10.3390/biom14050530

**Published:** 2024-04-28

**Authors:** Iogann Tolbatov, Paolo Umari, Alessandro Marrone

**Affiliations:** 1 Department of Physics and Astronomy, University of Padova, Via F. Marzolo 8, 35131 Padova, Italy; paolo.umari@unipd.it; 2 Dipartimento di Farmacia, Università “G d’Annunzio” di Chieti-Pescara, Via dei Vestini 31, 66100 Chieti, Italy; amarrone@unich.it

**Keywords:** diruthenium paddlewheel complexes, anticancer metallodrugs, protein metalation, substitution reactions, dual drugs

## Abstract

Metallodrugs are an important group of medicinal agents used for the treatment of various diseases ranging from cancers to viral, bacterial, and parasitic diseases. Their distinctive features include the availability of a metal centre, redox activity, as well as the ability to multitarget. Diruthenium paddlewheel complexes are an intensely developing group of metal scaffolds, which can securely coordinate bidentate xenobiotics and transport them to target tissues, releasing them by means of substitution reactions with biomolecular nucleophiles. It is of the utmost importance to gain a complete comprehension of which chemical reactions happen with them in physiological milieu to design novel drugs based on these bimetallic scaffolds. This review presents the data obtained in experiments and calculations, which clarify the chemistry these complexes undergo once administered in the proteic environment. This study demonstrates how diruthenium paddlewheel complexes may indeed embody a new paradigm in the design of metal-based drugs of dual-action by presenting and discussing the protein metalation by these complexes.

## 1. Introduction

Modern medicinal chemistry incorporates both purely organic and inorganic drugs [1]. The latter incorporate an important class of metallodrugs, which are often denominated as a separate group of therapeutic agents because of their unique features originating from the presence of a transition metal centre [2,3]. These features include structural variability, the attainability of various coordination numbers and redox states, as well as multiple ways to adjust their modes of action by attaching different ligands to the metal centre [4,5]. The thermodynamics and kinetics of their interactions with biomolecules are regulated by the suitable selection of both metal centres and metal ligands, thus modulating their electronic, electrostatic, lipophilic, and steric features [6,7,8]. 

The story of metallodrugs started with the serendipitous finding of the cytotoxic effect of cisplatin [9]; however, the presence of the adverse side-effects of the latter prompted researchers to look for novel scaffolds based on other transition metals [10,11]. Other metal centres, such as gold, copper, ruthenium, and rhodium, showcase geometries and redox properties differing greatly from those of platinum [12,13,14,15]. It is possible to accurately fine-tune the reactivity of these complexes by the correct choice of organic ligands [16]. Another strategy is to utilise therapeutic agents as ligands on a metal centre, thus combining two effects in the action of these complexes: the attack of a metal centre on its biomolecular targets and the action of cleaved ligands with medicinal properties. Complexes with such an amalgamation of anticancer properties of the metal centre and ligands are called dual-action metallodrugs because their employment permits the simultaneous employment of two routes of action, thus greatly enhancing the therapeutic impact. The synergistic effect of this technique results in improved drug administration, penetration of cellular membranes, as well as an enhanced possibility to solubilise [17,18,19]. 

**Figure 1:**
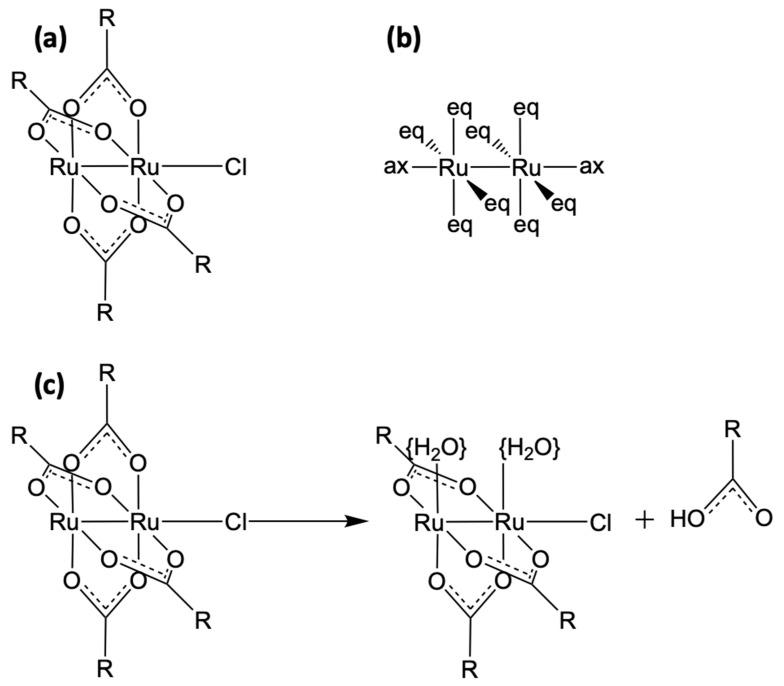
(**a**) The prototypical diRu tetraacetate paddlewheel complex, Ru_2_Cl(O_2_CR)_4_, with R = CH_3_; (**b**) axial and equatorial vacancies around the diRu core; (**c**) conceptual representation of the cleavage of a bridging ligand from the paddlewheel complex. If the bridging ligand is pharmacologically active, the diRu complex is a prodrug. Curly brackets were used to denote that water molecules are expected to be rapidly replaced by any μ-coordinating ligand available in the reaction environment.

Diruthenium complexes may include both the bimetallic Ru_2_^5+^core as well as bridging carboxylate ligands (Figure 1). The latter may be substituted by therapeutic agents, thus making these complexes dual-action drugs [20,21]. We foresee that these latter constructs may represent a new extension of the metallodrug paradigm by making the bimetallic paddlewheel moiety both a pharmacodynamic and a pharmacokinetic player. A multitude of various biologically active complexes have been used for this purpose. The employment of non-steroidal anti-inflammatory drugs (NSAIDs) has allowed for the combination of anti-inflammatory and anticancer properties as well as the enhancement of their cytotoxicities [22,23,24]. It has been shown that the severe side effects of NSAIDs diminish if these organic molecules are combined with a diruthenium core. Moreover, some diRu-NSAID paddlewheel complexes have exhibited high cytotoxicity [25,26,27]. The inclusion of peptides or fatty acids also yielded high biological activity against glioblastoma [20,28,29,30]. Indeed, the tetracarboxylate motif shields and facilitates the transportation of the bimetallic moiety. On the other hand, after cleavage, bridging therapeutically active ligands operate on their own. Thus, the accurate selection of bridging ligands with their characteristic electronic, steric, and electrostatic features permits the fine-tuning of the selectivities and behaviours of diRu paddlewheel complexes [31,32,33].

Prototypical diruthenium paddlewheel complexes, Ru_2_Cl(O_2_CR)_4_, are usually Ru_2_^5+^ species, although unstable tetracarboxylate Ru_2_^4+^ or Ru_2_^6+^ compounds can be obtained [34,35]. Nevertheless, the stability of different oxidation states depends on the ligands [36,37]. Bimetallic Ru_2_^5+^ cores are usually joined by chloride axial ligands in the solid state but, under physiological conditions, form discrete species at equilibrium, with water and chloride ligands at the axial positions, Ru_2_Cl(O_2_CR)_4_(OH_2_) and [Ru_2_(O_2_CR)_4_(OH_2_)_2_]^+^, and even [Ru_2_Cl_2_(O_2_CR)_4_]^–^ at high chloride concentrations [36,38]. Another important aspect is the possible deprotonation of axial water ligands at high pH levels, which produces a hydroxo instead of a water-bound form that may play a relevant role in the behaviour of these complexes in resemblance to the water/hydroxo equilibrium detected in other metallodrugs, such as cisplatin [9,11].

In this review, a compendium of experimental and theoretical studies, focused on diruthenium paddlewheel complexes, was reported by aiming to elucidate structure–activity relationships and paving the way to the development of newly designed bimetallic complexes.

## 2. Crystallographic Data

The first adduct of the paddlewheel tetrakis(acetato)chloridodiruthenium complex [Ru_2_Cl(O_2_CCH_3_)_4_] and the protein hen egg-white lysozyme (HEWL) was obtained and crystallised in 2014 [39]. The usage of ESI-MS and UV-Vis spectroscopy unequivocally proved the appearance of a stable metal–protein adduct with a preserved diruthenium core and two of four acetate ligands detached, whereas the structural data obtained via X-ray diffraction (XRD) analysis with a resolution of 2.1 demonstrated the formation of an adduct with Asp residues (Table 1).

**Figure 2:**
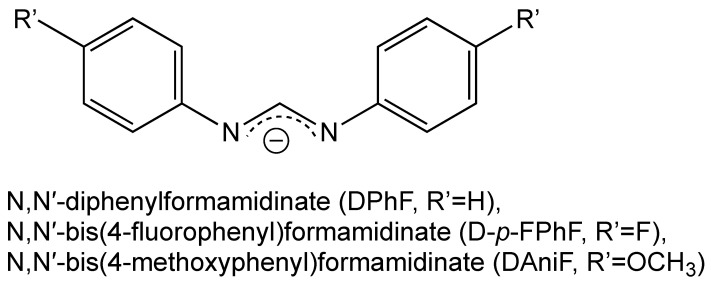
Selected bridging ligands.

To study the steric and charge impacts of the bridging ligands of the diruthenium paddlewheel complex on its cytotoxicity and ability to metalate proteins, a diruthenium complex decorated by two formamidinate ligands, [Ru_2_Cl(DPhF)_2_(O_2_CCH_3_)_2_], was synthesised as well as its two analogues with one formamidinate, [Ru_2_Cl(DPhF)(O_2_CCH_3_)_3_] and [Ru_2_(DPhF)(CO_3_)_3_] (DPhF^−^ = *N,N′*-diphenylformamidinate, Figure 2) [40]. The investigation of their binding with HEWL demonstrated that diruthenium complexes always conserve the intermetallic bond and yield stable adducts. Furthermore, the authors showed that the cytotoxic features of these complexes were directly correlated to the electronic features, steric hindrance, and lipophilicity of formamidinate ligands. The authors concluded that the most favourable covalent binding sites in HEWL are constituted by the side chains of Asp101 and Asp119. Moreover, they observed the propensity of diruthenium compounds to react via the substitution of their acetate-bridging ligands (Table 1).

The investigation of the reactivity of [Ru_2_Cl(D-*p*-FPhF)(O_2_CCH_3_)_3_] with HEWL substantiated its ability to metalate the protein, producing stable adducts of diruthenium complexes with Asp side chains via the loss of an acetate [41]. All the other acetates tend to stay with the D-*p*-FPhF ligand, being either cis or trans to the Asp side chain. These results corroborate that paddlewheel scaffolds remain stable after the protein metalation, with the Ru-Ru bond being preserved (Table 1).

The influence of various bridging equatorial ligands on the protein-attacking potential of diruthenium paddlewheel compounds was studied recently [42]. It was shown that the charge of these complexes is a key factor regulating their interaction with a protein. Various anionic complexes were synthesised, including [Ru_2_(CO_3_)_4_]^3−^, [Ru_2_(D-*p*-FPhF)(CO_3_)_3_]^2−^, and [Ru_2_(DAniF)(CO_3_)_3_]^2−^, as well as their acetate-based analogues [Ru_2_Cl(D-*p*-FPhF)(O_2_CCH_3_)_3_] and [Ru_2_Cl(DAniF)(O_2_CCH_3_)_3_] (bridging ligands in Figure 2). A multitude of experimental techniques—UV-Vis spectroscopy, X-ray crystallography, circular dichroism, and intrinsic fluorescence—were employed to investigate the interaction of these metal complexes with the protein HEWL. It was found that the charge potently affects their protein-binding properties. Complexes with highly negative charges tend to bind non-covalently, whereas the substitution of carbonate ligands with formamidinate alters their negative charge, thus inducing covalent binding. It was shown that [Ru_2_(O_2_CCH_3_)_4_]^+^ coordinates to the side chains of Asp residues, with one acetate ligand substituted by Asp, and shows a propensity towards exchanging other acetate ligands by water molecules. On the other hand, the complex [Ru_2_(D-*p*-FPhF)(O_2_CCH_3_)_3_]^+^ binds to HEWL via other solvent-accessible protein residue side chains—Asp101, Asp119, Asn19, Lys33, and Arg125—preserving the formamidinate bound to the diruthenium core. The authors juxtaposed the preferred metalations of [Ru_2_(CO_3_)_4_]^3−^ and [Ru_2_(O_2_CCH_3_)_4_]^+^. Whereas both complexes attack the Asp side chain, only the tetracarbonate complex might as well coordinate to Asn and the C-terminal tail, thus binding to the protein non-covalently. The complexes [Ru_2_(D-*p*-FPhF)(CO_3_)_3_]^2−^ and [Ru_2_(DAniF)(CO_3_)_3_]^2−^ exhibit the following behaviour: they stay non-covalently bound to the protein surface while the highly negative charge is preserved. As soon as a carbonate ligand is lost, either via substitution or via a straightforward release, their charge augments from −2 to −1, or even to positive values, thus enabling them to coordinate with the protein residue side chains, similar to cationic diruthenium compounds containing acetate ligands.

The structural data inherent in the binding of diRu paddlewheel complexes at protein targets clearly indicate that the Asp carboxylate is the preferred metalation site via the replacement of an equatorial ligand. The presence of diRu–protein adducts involving the majorly equatorial compared to the axial coordination of the metal fragment, for which only limited evidence has been reported [41], is probably because of the experimental conditions under which the targeted protein is typically incubated for a long time in buffered solutions of the paddlewheel complex. The formation of axial complexes in the early stage of the incubation cannot be ruled out.

Indeed, the axial coordination in these diRu complexes can be considered as being more labile compared to the equatorial coordination. The presence of water molecules coordinated at the axial position of these complexes testifies that these positions probably bear a harder character and, thus, give rise to weaker coordinative bonds.

The prominence of the equatorial coordination with targeted protein sites is also corroborated by the lack of evidence for the destabilisation of the Ru-Ru bond. The almost-exclusive targeting of Asp residues by diRu paddlewheel complexes is noticeable. Indeed, we envision that the side chain of Glu residues should evidence almost the same chemical reactivity as Asp carboxylate groups. Based on the absence of diRu paddlewheel complexes bound at Glu residues and the almost-exclusive Asp targeting, several hypotheses can be drawn. Probably, the higher abundance of Asp residues on the protein surface prompts their carboxylate groups to react with paddlewheel complexes and favours the occurrence of the equatorial ligands’ substitution on the external surface of the target. Such a hypothesis has been corroborated by experimental evidence locating diRu paddlewheel moieties mostly bound at Asp residues of solvent-accessible surfaces.

Another interesting point is revealing the lack of experimental evidence for the paddlewheel scaffold dismantling upon protein binding. Again, these data may reflect the almost-exclusive targeting of Asp residues that coordinate at two equatorial positions—we found only one evidence of the η^1^ coordination of the Asp carboxylate, thus stabilising the bimetallic moiety. Indeed, no elongation of Ru-Ru distances was detected in crystallised structures of diRu-based paddlewheel complexes with the protein HEWL.

## 3. Ligand Exchange Processes: A Thermodynamic Insight

Because the most common approach for investigating the behaviour of diRu paddlewheel complexes towards proteins is soaking for a long duration, most commonly up to 72 h [39,43]—in some cases, this time might be chosen to be as long as 7 days [44]—such long times result in the thermodynamic control over the substitution reactions in question. Taking into account that the substitution of an axial ligand in paddlewheel complexes is the most facile reaction energetically, as it includes the cleavage of only one bond, it is assumed that the coordination at the axial position happens initially. Such a coordination may occur on the surface of the protein or in the protein pocket reachable by the solvent. The substitution of an equatorial ligand in a paddlewheel complex is a more energetically demanding event, which necessitates the breaking of two metal–oxygen bonds. The overall reaction energy might be mitigated by the possibility of the later formation of two bonds between metals and either the attacking biomolecular nucleophile or the molecule of the solvent or substrate; however, the kinetic threshold for such a reaction involving the equatorial ligand is certainly quite high. This later reaction might happen further downstream, also involving the disruption of metal–metal bond.

**Figure 3:**
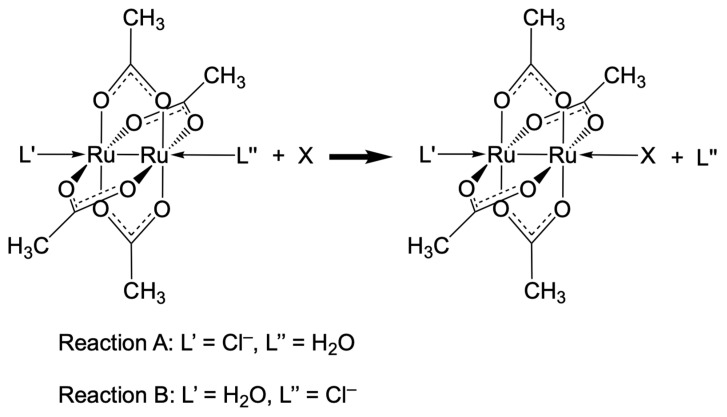
Scheme showing substitution reactions of axial ligands: water or chloride.

Several recent computational studies have focused on the Gibbs free energies for ligand exchange reactions between the complex [Ru_2_Cl(O_2_CCH_3_)_4_(OH_2_)] and simplified models of residue side chains (Figure 3) [45,46]. Indeed, the stability of this complex is dictated by the collective action of μ-bridging and axial ligands, which collaboratively affect the electronic/electrostatic character of the metallic core, thus altering the robustness of metal–carboxylate bonds. The employment of density functional theory (DFT), a workhorse of modern computational sciences [47], has permitted the investigation of a wide variety of bioinorganic and organometallic chemistry systems and, in particular, the production of accurate structures and reaction profiles for transition-metal-based anticancer complexes [48,49,50,51,52,53].

**Figure 4:**
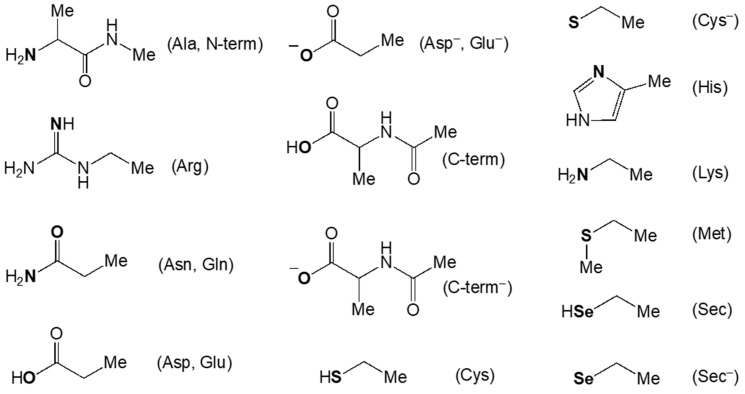
Simplified models of the protein residues investigated in [45,46]. The nucleophile atoms are in bold.

Two protonation states were utilised for aspartic acid, the C-terminal carboxylate, cysteine, and selenocysteine: a neutral one and an anionic one. For arginine, histidine, lysine, and the N-terminal, only the neutral state was employed because of their inability to coordinate in their protonated states (Figure 4).

In the case of the complex [Ru_2_Cl(O_2_CCH_3_)_4_(OH_2_)], its coordination to the nucleophile may occur via either the chloride or the water ligand exchange. The substitution of Cl^−^ showcases a high preference towards Cys^−^ and Sec^−^, with GFE values of −12.4 and −8.1 kcal/mol, respectively. The coordination of the diruthenium scaffold by residues Arg and Asp^−^ demonstrates a slight exergonicity (−3.8 and −0.8 kcal/mol, respectively), whereas all the other protein residues feature higher reaction enthalpies (1.0–25.1 kcal/mol).

The exchange of the axial ligand on the other side of the complex is much more facile, as it is corroborated by GFE values lower by 6-22 kcal/mol with respect to the exchange of the chloride (Table 2). This investigation has shown three distinct sets of protein residues, each demonstrating a diverse selectivity to coordinate with the complex. The reaction energies with Arg, Cys^–^, and Sec^–^ are the most favourable: −15.4, −23.5, and −18.0 kcal/mol, respectively. The reactions with Asp^–^, C-term^–^, His, Lys, Met, and N-term are all characterised by GFE energies between −13.4 and −5.0 kcal/mol. The third set incorporates all the other studied protein residues, for which the reactions have slightly exergonic or endergonic character.

These data demonstrate that the preferred way of the coordination for the complex [Ru_2_Cl(O_2_CCH_3_)_4_(OH_2_)] is the substitution of a water molecule at the axial position with Arg both because this reaction is thermodynamically favoured, with a GFE of −15.4 kcal/mol, and because Arg is ubiquitously found at the protein surface. The coordination to either Cys^−^ or Sec^−^, with GFE values of < 18.0 kcal/mol when they substitute axial water molecules and with GFE values of <−8.1 kcal/mol when they substitute chlorides, showcase a very high favourability; however, these residues are usually buried deep in proteins and, thus, their availability is subject to the conformational variability of the proteins.

## 4. Bimetallic Scaffold Stability

The structural analysis of the paddlewheel–protein residue adducts has shown that the coordination of a nucleophile at the axial position decreases the strength of M-M bonds unequivocally and, marginally, the strength of M–acetate bonds. Indeed, as it can be seen in Table 3, coordinations of Arg, Asp^−^, His, Lys, or Sec^−^ loosen M-M bond by 0.09, 0.04, 0.11, 0.11, or 0.14 Å, respectively. The highest destabilisation is observed for Sec^−^, which tends to form the strongest coordination with the soft metal centre Ru, being a soft ligand itself. The analysis of the NBO charges at both metal centres corroborates this idea. Indeed, as it can be seen from Table 3, coordinations to Arg, His, Lys, or Sec^−^ diminish the charge at the metal atom to which the nucleophile is coordinated, thus augmenting the charge at another metal centre. Thus, the charge distribution at the bimetallic core becomes asymmetrical. On the other hand, Asp^−^, which affects M-M bond to the least extent (only +0.04 Å), modifies the charges at the bimetallic core only marginally, preserving the symmetry of the charge distribution. The broken symmetry of the charge distribution between two metal centres, because of the coordination to Sec^–^ or N-containing Arg, His, or Lys, eventually advances the destabilisation of the bimetallic core, resulting in the subsequent downstream disintegration of the diRu paddlewheel complex. On the other hand, the eventual dismantling of the bimetallic moiety also depends on the nature of the equatorial bridging ligands; e.g., the presence of μ-coordinated formamidinate has been found to stabilise the bimetallic core [40,41,42].

## 5. Ligand Exchange Processes: A Mechanistic Insight

Reactions A and B were also investigated using the pseudo-molecular approach. Because the simultaneous axial coordination of both entering and leaving ligands is possible in the transition state, an interchange mechanism was hypothesised; thus, the activation barriers were calculated as the differences between the energies of the transition states and reactant adducts [46] (Table 4).

If the axial water molecule is substituted in the reaction by residues Arg and His, the activation energies are <10 kcal/mol, yielding a reaction characterised only by diffusion; i.e., as soon as these protein residues approach the axial water molecule, the exchange takes place, and the protein metalation immediately occurs. Residues Cys, Lys, and Sec also substitute the water molecule under physiological conditions, their activation energies are within the interval 10–12 kcal/mol. On the other hand, Cys^−^ and Sec^−^ react with slightly higher activation costs of 17.6 and 19.5 kcal/mol, respectively, which is explained by their anionic character and the neutral charge of the deaquated paddlewheel complex.

The overall reactivity is inferior in the case of the chloride substitution (reaction B) with respect to the water ligand exchange; nevertheless, the order of the activation free energies follows the same pattern. The energy barriers for the substitutions of Arg and His are the lowest, 18.2 and 18.5 kcal/mol, respectively, while Cys, Lys, and Sec reveal activation barriers of 20.4–22.5 kcal/mol. Again, anionic nucleophiles Cys^−^ and Sec^−^ showcase barriers of 24.7 and 26.5 kcal/mol, respectively. These values agree well with reaction half-lives of several hours under physiological conditions.

The results of the computational studies on axial substitutions in Ru_2_Cl(O_2_CCH_3_)_4_(OH_2_)] show that (a) the substitution of water is the more feasible route and (b) Arg and His are the most likely targets in proteins.

## 6. Concluding Remarks: Axial or Equatorial Coordination?

The reported studies corroborate the idea that diruthenium paddlewheel complexes (axial)_2_Ru_2_(equatorial)_4_, for which (H_2_O)(Cl)Ru_2_(µ-O_2_CCH_3_)_4_ is a prototypical example, initially coordinate at the axial position via water exchange with a biomolecular nucleophile, whereas equatorial coordination with protein residues (and, likely, other possible nucleophiles) happens only at a relatively later time. The structural analysis of the bimetallic scaffold as well as the charge distribution analysis demonstrate that intermetallic bonds are substantially weakened by axial coordination. These observations considerably impact our understanding of the adequate design of diruthenium-core-based metallodrugs.

We should also notice that real systems incorporate not only metal scaffolds and single attacking nucleophiles but also supplementary molecules, which stay on the solvent-accessible surfaces of metallodrugs after conventional binding positions are taken. These surplus molecules may be biomolecular nucleophiles or solvent or substrate molecules, which may stabilise metallodrug–nucleophile adducts via charge, dipole, or hydrogen-bond interactions. That is why we should consider the non-covalent coordination of the surrounding moieties not only as a stage antecedent to covalent binding but also as an outcome of all the possible binding sites around metal centres being complete. Herein, the charge of the metal complex is envisioned to play a major role.

The employment of Ru_2_^5+^ paddlewheel complexes in the design of novel dual drugs can be considered as a new paradigm of “metals in medicine”. The diRu metal scaffold has been found to stably coordinate bidentate xenobiotics that can be delivered in target tissues and released via exchange with endogenous nucleophiles. The fine-tuning and handling of such metal-based molecular devices are necessarily dependent on a full understanding of the chemical events underlying ligand exchange in these bimetallic scaffolds.

In this report, the body of information harvested by either experiments or calculations was presented and discussed to assess the potential use of Ru_2_^5+^ paddlewheel complexes as dual-acting drugs and form the basis of plausible structure–activity relationships.

In particular, the joint experimental/theoretical picture of protein metalation by diruthenium paddlewheel complexes evidenced the early involvement of axial coordination, which allowed for the anchoring of the target. We hypothesised that the articulation of several thermodynamically controlled ligand exchanges occurring at the axial position of the diRu scaffold may increase the residence time of the metal complex on the protein target, thus eventually permitting the less-favoured substitution of equatorial ligands.

The specific combination of axial and equatorial ligand exchange events that eventually determines the pharmacodynamic destiny of Ru_2_^5+^ paddlewheel complexes depends not only on the nature of coordinative bonds involving both axial and equatorial ligands but also on their chemical nature and their possible non-covalent interactions with the target. Therefore, pendant groups not directly involved in ligand exchange processes could be usefully designed to fine-tune either pharmacokinetics and/or tissue selectivity.

Overall, the present review evidenced how Ru_2_^5+^ paddlewheel complexes may indeed represent a new paradigm in the development of metal-based dual-acting agents by providing structural bases for their design and development.

## Figures and Tables

**Table 1:** Coordinative bond distances detected in crystallised structures of diRu-based paddlewheel complexes with the protein HEWL. (No data exist in the RCSB database for the usage of other proteins.) When several equivalent bonds are present, the reported value is the average. All the distances are in ångströms. μ-O,O-ligands: Asp = β-carboxylate, OAc = acetyl carboxylate, COO = buffer carboxylate, CO_3_ = carbonate ion, Leu = C-terminal carboxylate, and succ = succinate; μ-N,N’-ligands: form = *N,N’*-diphenylformamidinate (DPhF), *N,N’*-bis(4-fluorophenyl)formamidinate (D-*p*-FPhF), or *N,N’*-bis(4-metoxy-phenyl)formamidinate (DAniF); N-ligands: Arg = ε-guanidine and Lys = ε-amine.

Complex (Axial) Ru_2_ (Equatorial)	Adduct (Axial) Ru_2_ (Equatorial)	H_2_O	Ru	H_2_O	OAc	COO	CO_3_	Asp	Asp *	succ	Leu	form	Arg/ Lys	PDB
(Cl)Ru_2_(μ-O_2_CCH_3_)_4_	[(H_2_O)_2_Ru_2_(μ-O_2_CCH_3_)_2_(OH_2_)_2_]^3+^	2.74	2.31	2.64	2.01	-	-	-	-	-	-	-	-	4ooo, [39]
	[(H_2_O)(Cl)Ru_2_(μ-O_2_CCH_3_)_2_(OH_2_)_2_]^2+^	2.58	2.24	1.84	2.01	-	-	2.04	-	-	-	-	-	4ooo, [39]
(Cl)Ru_2_(DPhF)(O_2_CCH_3_)_3_	(H_2_O)(Cl)Ru_2_(DPhF)(μ-O_2_CCH_3_)(succ)(Asp)	2.61	2.30	-	2.06	-	-	-	2.09, 2.57, 2.71	2.71	-	2.02	-	8ph7, [40]
	[(H_2_O)(Lys)Ru_2_(DPhF)(μ-O_2_CCH_3_)(succ)_2_]^+^	2.06	2.27	-	2.04	-	-	-	-	1.92	-	2.02	2.47	8ph7, [40]
(Cl)Ru_2_(DPhF)_2_(O_2_CCH_3_)_2_	[(H_2_O)Ru_2_(DPhF)_2_(μ-O_2_CCH_3_)(Asp)]^+^	2.38	2.29	-	-	2.09	-	2.03	-	-	-	2.05	-	8ph5, [40]
	[(H_2_O)_2_Ru_2_(DPhF)_2_(μ-O_2_CCH_3_)(Asp)]^+^	2.40	2.33	-	-	2.08	-	2.10	-	-	-	2.04	-	8ph5, [40]
(Cl)Ru_2_(D-*p*-FPhF)(O_2_CCH_3_)_3_	[(H_2_O)_2_Ru_2_(D-*p*-FPhF)(μ-O_2_CCH_3_)_2_]^2+^	2.00	2.25	-	2.05	-	-	-	-	-	-	2.02	-	8bpj, [41]
	[(H_2_O)_2_Ru_2_(D-*p*-FPhF)(μ-O_2_CCH_3_)_2_(Asp)]^+^	2.57	2.28	-	-	2.03	-	2.15	-	-	-	2.01	-	8bpu, [41]
(Cl)Ru_2_(DAniF)(O_2_CCH_3_)_3_	[(H_2_O)Ru_2_(DAniF)(μ-O_2_CCH_3_)_2_(Asp)]^+^	2.12	2.28	-	2.05	-	-	2.11	-	-	-	2.04	-	8pfv, [42]
[Ru_2_(CO_3_)_4_]^3−^	[Ru_2_(CO_3_)_3_(Leu)]^2−^	-	2.29	-	-	-	2.16	-	-	-	2.25	-	-	8pfu, [42]
[Ru_2_(DPhF)(CO_3_)_3_]^2−^	[(H_2_O)Ru_2_(DPhF)(COO)(CO_3_)_2_]^−^	2.18	2.29	-	-	2.03	2.04	-	-	-	-	2.04	-	8ph6, [40]
	[(H_2_O)Ru_2_(DPhF)(Asp)(COO)(Arg)]^2+^	2.30	2.29	-	-	2.05	-	2.11	-	-	-	2.05	2.10	8ph6, [40]
	[(H_2_O)Ru_2_(DPhF)(CO_3_)_3_]^2−^	2.17	2.26	-	-	-	2.05	-	-	-	-	2.05	-	8ph6, [40]
	[(H_2_O)Ru_2_(DPhF)(Asp)(COO)(O_2_CCH_3_)]^+^	2.35	2.27	-	2.02	2.07	-	2.04	-	-	-	2.04	-	8ph6, [40]
[Ru_2_(D-*p*-FPhF)(CO_3_)_3_]^2−^	[Ru_2_(D-*p*-FPhF)(CO_3_)(OH_2_)_2_]^2+^	-	2.26	2.04	-	-	2.05	no	-	-	-	2.01	-	8pft, [42]
	[Ru_2_(D-*p*-FPhF)(Asp)(CO_3_)(OH_2_)_2_]^+^	-	2.29	2.03	-	-	2.04	2.25	-	-	-	2.02	-	8pft, [42]
[Ru_2_(D-*p*-FPhF)(CO_3_)_3_]^2−^	[(H_2_O)_2_Ru_2_(D-*p*-FPhF)(Asp)(CO_3_)(OH_2_)_2_]^+^	2.26	2.30	2.02	-	-	2.04	2.12	-	-	-	2.02	-	8pfx, [42]
[Ru_2_(DAniF)(CO_3_)_3_]^2−^	[(H_2_O)_2_Ru_2_(DAniF)(CO_3_)_3_]^2−^	2.55	2.29	-	-	-	2.04	-	-	-	-	2.04	-	8pfw, [42]
	[(H_2_O)Ru_2_(DAniF)(Asp)(CO_3_)_2_]^−^	2.36	2.28	-	-	-	2.04	2.06	-	-	-	2.03	-	8pfw, [42]
	[(H_2_O)Ru_2_(DAniF)(CO_3_)_3_]^2−^	2.31	2.27	-	-	-	2.06	-	-	-	-	2.03	-	8pfw, [42]
	average distance	2.35	2.28	2.11	2.03	2.06	2.06	2.10	2.46	2.32	2.25	2.03	2.29	

* This column features Ru-O(Asp) bonds in cases when Asp is interacting with the metal centre(s) only with one oxygen.

**Table 2:** Gibbs free energies for reactions of the substitution of axial ligands in [Ru_2_Cl(O_2_CCH_3_)_4_(OH_2_)] [45] (in kcal/mol).

Protein Site	Substitution of Axial Ligand	
	Water (Reaction A)	Chloride (Reaction B)
Arg	−15.4	−3.8
Asn(N)	4.5	18.9
Asn(O)	−0.7	11.5
Asp	−0.1	18.2
Asp^–^	−10.0	−0.8
C-term	1.5	25.1
C-term^–^	−5.0	1.0
Cys	−5.0	12.9
Cys^–^	−23.5	−12.4
His	−10.4	2.5
Lys	−13.4	1.6
Met	−5.8	14.1
N-term	−7.7	7.1
Sec	−4.4	12.2
Sec^–^	−18.0	−8.1

**Table 3:** Effects of the axial nucleophiles on the metal centres in the complex [Ru_2_Cl(O_2_CCH_3_)_4_(OH_2_)] [45]. Distances are in ångströms.

Axial Ligand X	Distance	NBO Charges	
	M-M Distance	Ru1	Ru2
Water1	2.30	1.07	1.05
Arg	2.39	0.87	1.19
Asp^–^	2.34	1.02	1.06
His	2.41	0.88	1.21
Lys	2.41	0.89	1.19
Sec^–^	2.44	0.75	1.03

**Table 4:** Activation free-energy values for reactions A and B [46] (in kcal/mol).

Protein Site	Substitution of Axial Ligand	
	Water (Reaction A)	Chloride (Reaction B)
Arg	4.5	18.5
Cys	11.1	20.4
Cys^–^	15.7	22.8
His	7.6	18.2
Lys	10.9	22.5
Sec	11.9	20.4
Sec^−^	17.6	24.6

## Data Availability

The data are contained within the article.
